# Navigating Unique Diagnostic and Therapeutic Challenges in Premenstrual Dysphoric Disorder: A Case Report

**DOI:** 10.7759/cureus.63238

**Published:** 2024-06-26

**Authors:** Aateqa Hashmi, Fariha Bangash, Luba Leontieva

**Affiliations:** 1 Psychiatry, State University of New York Upstate Medical University, Syracuse, USA

**Keywords:** case report, interpersonal conflicts, mood lability, depression, premenstrual symptoms

## Abstract

Premenstrual dysphoric disorder (PMDD) is a disabling form of premenstrual syndrome affecting females of reproductive age in the premenstrual period. The presentation may vary from severe mood lability to extreme attempts to end life, usually within a week before menstruation resulting in considerable stress, functional impairment, and interpersonal conflicts. We present an interesting case of a 19-year-old sexually active girl who presented with a polysubstance overdose owing to her cyclical episodes of severe mood symptoms including irritability and uncontrolled aggression. Detailed history and thorough examination raised suspicion of PMDD which was confirmed on prospective symptom charting for two menstrual cycles as described by the Diagnostic and Statistical Manual of Mental Disorders, Fifth Edition (DSM-5) diagnostic criterion. After the establishment of diagnosis, the patient was started on selective serotonin reuptake inhibitors to target PMDD symptoms along with oral contraceptive pills for birth control which showed marked improvement in her overall condition. We herein discuss multiple diagnostic and therapeutic challenges that limit correct diagnosis and timely management of PMDD, especially in the adolescent age group.

## Introduction

Premenstrual dysphoric disorder (PMDD) is a severe and disabling form of premenstrual syndrome (PMS) that affects up to 3-8% of women [1]. These symptoms occur in menstruating women of the reproductive age group only hence not occurring before menarche, during pregnancy, or post menopause. PMDD has no evidence-based ovarian hormone as a biomarker for diagnosis [2]. PMDD is a cluster of affective and somatic symptoms that cause significant distress or malfunctioning classically in the week before the onset of menstruation, after which they subside to minimal or absent.

## Case presentation

A 19-year-old nulliparous woman presented in the emergency room (ER) with an intentional polysubstance overdose of unknown quantities of ibuprofen, acetaminophen, and dextromethorphan/pseudoephedrine in an attempt to end her life. There was no evidence of hepatitis, acidosis, or other toxidrome. She was thoroughly evaluated on voluntary inpatient psychiatric admission due to impulsivity and persistent suicidal ideation. Her medical history included asthma and her surgical history was unremarkable. She had no allergies. She was a non-smoker and consumed alcohol socially. She had a positive family history of her father with alcoholism, a paternal uncle with bipolar and substance use disorder, and a paternal cousin with bipolar disorder. She had no history of sexually transmitted infections or abnormal cervical cytology. Her menstrual flow was normal.

She reported becoming more argumentative and emotionally labile, and various episodes of uncontrollable and erratic behavior for the last six months. Her gynecological history included irregular menstrual cycles occurring once or twice a year since her menarche at 11 years old. She tried birth control pills for her irregular menstrual cycles which helped with the regulation of her cycles but she started experiencing depressive and irritable mood episodes and discontinued the pills. Six months ago, she became sexually active and started levonorgestrel-ethinyl estradiol for birth control. Her cycles became regular but her "mood episodes" began again. Two days before presenting in the ER, she started drospirenone/ethinyl estradiol for birth control and reported various episodes of irrational anger that she couldn’t control. The patient presented a history of severe physical and psychological symptoms related to her menses. She described that "I am not myself doing crazy things" and described depersonalization and derealization during the outbursts. One to two weeks before her menstruation, she would have various episodes of uncontrollable irritability and anger towards her mother and boyfriend. Some of the episodes included sending texts to them expressing anxiety that they don’t love her; throwing her phone shattering it into pieces; cutting herself to feel relief but it didn’t help her, and ingesting several pills in her dorm room because of feeling overwhelmed with her upcoming midterms; and returning to her mother and boyfriend with slurred speech while they were waiting for her outside her dorm. After these outbursts were over, she would become regretful and apologetic for her behavior. She also reported low mood, anhedonia, increased sleep, difficulty concentrating on school assignments, fatigue, and severe abdominal cramping during her premenstrual period which would abate three to five days after the onset of menstruation. Her fluctuating mood symptoms severely affected her normal functioning and interpersonal relationships. She had started taking therapy sessions in her early teenage years due to the physical and verbal abuse endured by her alcoholic father. Coping mechanisms taught during her therapy like deep breathing didn't help her regain control during the episodes. Lifestyle modifications recommended by her primary care physician including reduced consumption of refined carbohydrates and artificial sweeteners, reduced caffeine intake, and cognitive behavioral therapy were ineffective.

Her abdomen was soft and non-tender. To rule out thyroid disorder, laboratory investigations including a full blood count and thyroid function tests were performed and found normal. She was referred to reproductive psychiatry and gynecology for further evaluation which appeared unremarkable. Speculum and bimanual examination confirmed a normal vulva, vagina and cervix. Previous pelvic ultrasound scans were normal. Her body mass index (BMI) was reported to be 42.43. Lab testing revealed her acetaminophen levels were 105 ug/mL (normal levels: 10-30 ug/mL).

She maintained a diary of her symptoms and found a clear cyclical pattern related to her menstruation. Her mood symptoms increased dramatically in the premenstrual period, followed by a sense of relief after periods started. The Daily Record of Severity of Problems (DRSP) score, which is used for diagnosis and assessment of the Diagnostic and Statistical Manual of Mental Disorders, Fifth Edition (DSM-5) PMDD, was completed for two menstrual cycles and the woman scored highly indicating extreme symptoms. At least five symptoms (affective lability, irritability, anger, depressed mood, and lethargy) were present in the final week before the onset of her menses. Her symptoms started to improve within a few days after the onset of her menses and became minimal or absent in the week post-menses. Her symptoms were not attributable to the physiological effects of a substance (e.g. drug abuse, medication, or other treatment) or another medical condition (e.g. hyperthyroidism).

Her history and both physical and psychological symptoms correlated with her symptom diary, and the questionnaire suggested the diagnosis of PMDD. There was no underlying depressive or anxiety disorder. Conservative management using an integrated holistic approach in combination with pharmacological treatment was discussed.

Pregnancy was excluded prior to starting treatment. As the detailed evaluation of the patient was being carried out and the patient reported a family history of bipolar disorder, she was initiated on 25 mg lamotrigine for mood stabilization instead of a selective serotonin reuptake inhibitor (SSRI) as the latter can precipitate mania in bipolar disorder. After bipolar was ruled out, lamotrigine was discontinued and the patient was started on sertraline 50 mg one tablet daily to target her PMDD symptoms. She was counseled about its side effects including nausea, headache, dizziness, and dry mouth. Drospirenone/ethinyl estradiol 3-0.02 mg one tablet daily was prescribed for birth control. The patient was discharged on this treatment plan.

On subsequent outpatient follow-up visits, our patient expressed great emotional relief, and "stability" in her own words, following her diagnosis of PMDD. She finally felt "listened to" and felt motivated to start working towards her dream of getting an education degree and becoming a middle school English teacher. Her positive response to treatment with SSRIs provided clinical evidence that her premenstrual mood disruption was due to a malfunctioning serotonergic system. The DRSP questionnaire following her treatment with SSRIs indicated minimal symptoms, and she reported an improvement in her quality of life.

## Discussion

PMDD was introduced into the American Psychiatric Association's DSM-5 in 2013. WHO included PMDD in the International Classification of Diseases and Related Public Health Problems (ICD-11) in 2019 as a disorder of the reproductive tract. This simultaneous inclusion of PMDD, as a medical disorder in ICD-11 and a psychiatric disorder in DSM-5, highlights the overlapping somatic and affective symptoms.

Approximately between 3% and 8% of women meet the diagnostic criteria for PMDD which is a disabling mental health condition affecting females of reproductive age group. Etiology is thought to be multifactorial including biological, psychological, environmental, and social factors. Before the onset of menstruation, females with PMDD experience multiple mood and physical symptoms which are hypothesized to occur owing to altered sensitivity to physiological hormonal fluctuations. These symptoms arising in a female, if ignored and not treated, may cause significant deterioration of interpersonal relationships and quality of life, sometimes resulting in women attempting to or ending their own lives like our patient who presented with polysubstance overdose. Different hormones are postulated to have a role in causing mood symptoms in the premenstrual period: allopregnanolone can cause impulsivity, labile mood, aggression, or irritability; estrogen can cause neuronal excitability while progestins are inhibitory; dysregulated serotonergic system can play a role as well as decreased serotonergic activity has been noted in the premenstrual phase that responds to SSRIs as first line of PMDD treatment causing marked improvement in mood symptoms.

Our patient fulfilled the PMDD criteria of having at least 5 out of 11 stipulated symptoms identified in the DSM-5, one of which must include mood, behavioral, or physical symptoms with substantial stress or functional impairment. Confirming symptoms by prospective patient mood charting for at least two menstrual cycles was followed as recommendations. DRSP as a reliable tool is applied for at least two symptomatic cycles to confirm the timing of mood symptoms that our patient followed, hence confirming her PMDD diagnosis. A thorough history was taken from our patient to rule out the possibility of premenstrual symptoms as an exacerbation of an underlying disorder. Also, though PMDD can be superimposed on an axis I or II disorder, this was not the case in our patient either.

Establishing PMDD diagnosis and implementing appropriate management relies on the knowledge and clinical expertise of the physician and poses a diagnostic and therapeutic challenge. A study indicates that females with PMDD presenting with mood symptoms have a high chance of falling victim to clinical misjudgment and getting erroneously diagnosed with other serious psychiatric disorders, which limits further exploration resulting in professionals missing critical opportunities to correctly diagnose and treat PMDD [1].

Understanding context is very important for proper diagnosis and treatment, so we spent time with our patient, took a detailed history, taking the time to understand our patient’s perspective during these complex periods leading us to a more accurate diagnosis and confident treatment plan [3].

During the evaluation, if a patient does not report a symptom-free time period during the menstrual cycle, considering other differential diagnoses becomes important including thyroid disorders, substance use, eating disorders, or other mood disorders including major depression, bipolar disorder, generalized anxiety disorder, panic disorder, all of which were ruled out in our patient.

Management options for PMDD include psychotropic agents (SSRIs) and ovulation suppression agents (combined oral contraceptive pill (COCP) or gonadotropin-releasing hormone (GnRH) agonists). The recommended doses of SSRIs include sertraline 50-100 mg, fluoxetine 20 mg, escitalopram 10-20 mg, and paroxetine 10-20 mg. Our patient was discharged on sertraline 50 mg one tablet daily targeting her PMDD mood symptoms and was counseled about its side effects including nausea, headache, dizziness, and dry mouth. Drospirenone/ethinyl estradiol of 3 mg/0.02 mg one tablet daily was prescribed for birth control. On her outpatient follow-up visits, she expressed marked emotional relief and improvement in her aggression and irritability symptoms helping her to maintain stability in her life.

An important thing to consider in PMDD women wishing to get pregnant is that they ought to be educated about spontaneous discontinuation of PMDD symptoms during pregnancy, making use of SSRIs before and during pregnancy unnecessary and even risky due to a small unproven association with congenital defects such as anal atresia, hypospadias, clubfoot, gastroschisis, or an omphalocele. Moreover, uninterrupted dosing of oral contraceptive pills (OCPs) without allowing conventional hormone-free periods to produce withdrawal menses might be more efficacious than traditional dosing regimens. Spironolactone can reduce abdominal bloating, swelling, breast discomfort, and mood symptoms while SSRIs can improve mood symptoms including irritability, dysphoria, and depression, and physical symptoms like bloating, appetite changes, and breast tenderness much more promptly than they treat depression. Ramelteon can be effective in the treatment of residual sleep disturbances (hypersomnia) in PMDD patients [4].

If PMDD diagnosis is still ambiguous after two months of prospective symptom charting, GnRH agonists like leuprolide can aid in the establishment of diagnosis but owing to the significant side effect burden; they should be reserved for severe refractory cases to OCPs and SSRIs. Other treatments may include lifestyle modifications, cognitive behavioral therapy, or dietary supplementation with calcium, vitamin E, and vitamin B6. Rarely, bilateral oophorectomy (without removal of the uterus) with a preoperative trial of GnRH agonists and subsequent progesterone hormonal replacement therapy may be indicated in severe refractory cases, but this treatment regimen can predispose to a risk of progesterone-induced premenstrual disorder and relapse of PMS like symptoms [2].

Combining cognitive behavioral therapy psychotherapy and pharmacotherapy [5] to reach the patient's core beliefs and meet the expectations of the patient and her family for early relief from irritable and aggressive mood symptoms was an optimal choice guiding us through the treatment process.

Whenever our patient tried to use OCPs for irregular cycles or birth control, her mood symptoms would get more disruptive and out of control. A randomized clinical trial revealed that the use of an OCP can aggravate mood swings and irritability [6]. Especially the adolescent age group like our patient are more likely to report increased crying, hypersomnia, and eating problems while taking OCPs [7] getting their first diagnosis of major depressive disorder and hence requiring treatment with antidepressants, suggesting depression is an important potential adverse effect of OCPs [8].

Females with a history of emotional adverse effects when using combined contraceptives reported significant mood deterioration. Exogenous progestins increase levels of monoamine oxidase more than natural progesterone does, which causes decreased serotonin concentrations and thus potentially causes irritability and depression. A recent double-blind study revealed the appearance of subclinical depressive symptoms in females of the intervention group when they were randomized to a goserelin (GnRH agonist) implant or placebo. The depressive symptoms were directly related to the decrease in estradiol concentrations [8].

These OCPs-induced worsening of mood symptoms can adversely affect already suffering patients like our patient was struggling with PMDD. With correct diagnosis of PMDD and adding SSRI to her medications provided significant improvement in her mood, quality of life, and interpersonal conflicts. Identifying or ruling out PMDD in women of reproductive age with cyclical mood changes and physicians and psychiatrists being aware of its diagnostic and therapeutic challenges is important for correct diagnosis and timely management of PMDD.

Treatment of PMDD in women with other medical or psychiatric disorders becomes especially very challenging due to the overlap of presenting mood symptoms and available treatment options for coexisting comorbidities. A case report stated a 17-year-old girl who after sustaining a severe traumatic brain injury (TBI) developed symptoms of severe depression surrounding her menses, was diagnosed with PMDD, and ultimately required antipsychotics for symptom resolution [9]. Another case report explored a 37-year-old woman diagnosed with premenstrual exacerbation of schizophrenia or coexisting PMDD with schizophrenia. Treatment required with maximal doses of SSRIs and COCPs produced intolerable side effects and GnRH agonists, though effective, were not suitable for long-term use. Alternatively, dienogest (44th generation progestin) drastically improved premenstrual mood symptoms and irritability suggesting its safety for long-term use in PMDD patients with schizophrenia [10].

Similarly, a 45-year-old Caucasian female diagnosed with bipolar disorder with psychotic features, PMDD, panic disorder, and repeated occurrence of suicide attempts and self-harming behavior reported limited gains through standard medicinal treatment and showed dramatic clinical improvement after introduction of lifestyle interventions including dietary change, coffee enemas, dry-skin brushing and meditation, suggesting lifestyle modifications as the first-line therapy in PMDD patients with comorbidities when medications fail [11]. Another case report of a young woman diagnosed with polycystic ovarian syndrome (PCOS) described three episodes of premenstrual mixed mood and psychotic symptoms making already challenging PMDD diagnosis more difficult [12].

Working with adolescents diagnosed with PMDD demands more thoughtful management to maintain compliance. Asking an adolescent to keep track of symptoms using a menstrual calendar might make her not come back as evidence suggests that adolescents are less likely to ask for professional help even when it is needed [6]. It can cause delays in follow-up visits or seeking professional help not at all. Another difficult aspect in diagnosing PMDD in adolescents is an overlap of mood swings and aggression related to PMDD with sudden mood changes which are attributed to the pubertal struggles of adolescence. The physician may interpret an emotional lability as normal for age considering pubertal changes in adolescents, possibly causing a delay in correct diagnosis. Another exception in adolescents is that ovarian cycles are often irregular due to anovulatory cycles and are not predictable like that of an adult female. With the maturation of the hypothalamus-hypophysis-ovarian axis, the transition from irregular to regular menstrual cycles may vary for every adolescent girl. This raises the question of whether thorough history taking or the use of a screening tool can replace the time spent collecting two months of data, thereby avoiding unnecessary delays in diagnosing and treating adolescents with irregular cycles.

## Conclusions

Correct diagnosis of PMDD poses a challenge for psychiatrists due to its overlap with other mood disorders and symptom charting protocols. Identifying or ruling out PMDD in women of reproductive age with cyclical mood changes requires updated medical knowledge of physicians and psychiatrists so they are aware of its dynamic diagnostic and therapeutic challenges. This may ensure correct diagnosis and timely management of PMDD so patients don’t suffer in silence and may live without huge impairment to their quality of life.
